# A new structural class of bacterial thioester domains reveals a slipknot topology

**DOI:** 10.1002/pro.3478

**Published:** 2018-09-25

**Authors:** Ona K. Miller, Mark J. Banfield, Ulrich Schwarz‐Linek

**Affiliations:** ^1^ Biomedical Sciences Research Complex and School of Biology University of St Andrews St Andrews KY16 9ST United Kingdom; ^2^ John Innes Centre, Department of Biological Chemistry Norwich Research Park Norwich NR4 7UH United Kingdom

**Keywords:** bacterial surface proteins, TIE proteins, thioester domains, crystal structures, *Bacillus anthracis*, *Staphylococcus aureus*, *Enterococcus faecium*

## Abstract

An increasing number of surface‐associated proteins identified in Gram‐positive bacteria are characterized by intramolecular cross‐links in structurally conserved thioester, isopeptide, and ester domains (TIE proteins). Two classes of thioester domains (TEDs) have been predicted based on sequence with, to date, only representatives of Class I structurally characterized. Here, we present crystal structures of three Class II TEDs from *Bacillus anthracis*, vancomycin‐resistant *Staphylococcus aureus*, and vancomycin‐resistant *Enterococcus faecium.* These proteins are structurally distinct from Class I TEDs due to a β‐sandwich domain that is inserted into the conserved TED fold to form a slipknot structure. Further, the *B. anthracis* TED domain is presented in the context of a full‐length sortase‐anchored protein structure (BaTIE). This provides insight into the three‐dimensional arrangement of TIE proteins, which emerge as very abundant putative adhesins of Gram‐positive bacteria.

## Introduction

Adhesion of microbes to target molecules is a critical step in colonization and maintenance of infection. To that end, bacteria express and display a variety of surface proteins, which are subjected to a number of environmental stresses and must, therefore, possess remarkable inherent stability. Gram‐positive bacteria employ intramolecular isopeptide bonds^1^ and ester bonds^2^ to stabilize their surface‐associated proteins. Internal thioester bonds, formed between Cys and Gln side‐chains and first identified in the second thioester domain (TED) of the *Streptococcus pyogenes* minor pilin Cpa,^3^ play a negligible role in protein stability,^4^ but are suggested to mediate covalent bacterial adhesion. Identification of a single Lys side‐chain on the Aα chain of fibrinogen as the physiological receptor of the *S. pyogenes* SfbI TED, which reacts with the bacterial thioester to form an intermolecular isopeptide bond, supports this hypothesis.^5^

Covalent binding of bacteria to host tissues represents a rare case of convergent evolution in protein chemistry,^6^ as the only other known proteins containing intramolecular thioesters are members of the complement family, which function by covalently tagging pathogens for phagocytosis. However, bacterial TEDs and complement proteins are sequentially and structurally unrelated. Complement proteins such as C3 and C4 have a multi‐domain architecture and require proteolytic activation to expose their thioester, which subsequently reacts indiscriminately with nucleophiles, for instance, water or nucleophilic moieties on bacterial surfaces.^7^ Bacterial TEDs characterized to date comprise only a single domain, and as demonstrated by SfbI‐TED binding to fibrinogen, show high selectivity, and do not require proteolytic activation.^5^ It remains unknown how access to and reactivity of bacterial thioesters are regulated.

TEDs are predicted at the distal end of a large number of surface proteins from Gram‐positive bacteria, and the name TIE (thioester, isopeptide, ester) proteins have been introduced for this family.^5^ Multiple sequence alignment of a number of experimentally characterized and predicted TEDs suggested they could be divided into two structural classes, however a structural basis for this distinction was lacking. Class I, including both Cpa‐TEDs and SfbI‐TED, appears to possess an N‐terminal indel of 15–20 amino acids absent in Class II. In contrast, Class II appears to possess an extended C‐terminal indel absent in Class I, resulting in an approximately 30% mass increase. In both classes, bond‐forming Cys and Gln residues are found in a [YFL]CΦζ amino acid motif (where Φ is any hydrophobic and ζ is any hydrophilic residue) and a weak ΦQζΦΦ motif, respectively. A third motif, TQXXΦWXΦXζ, has also been identified in all TEDs predicted to date, and while no definitive explanations as to its function have been determined, its conserved Gln and Trp residues are not essential for thioester bond formation.^8^ Despite the presence of definable motifs, the only residues universally conserved in TEDs are the bond‐forming Cys and Gln.

Here we present the Class II TED fold, structurally conserved across three Gram‐positive genera, despite considerable sequence divergence. Structures of *Bacillus anthracis*, vancomycin‐resistant *Staphylococcus aureus*, and vancomycin‐resistant *Enterococcus faecium* TEDs show a head domain with Class I topology extended by a β‐sandwich; the latter contributes the thioester bond‐forming Gln via a β‐hairpin insertion into the head domain. The structure of the *B. anthracis* TED was determined in the context of the mature, full‐length TIE protein, and reveals how TEDs are presented away from the bacterial cell surface to engage receptors.

## Results and Discussion

**Figure 1:**
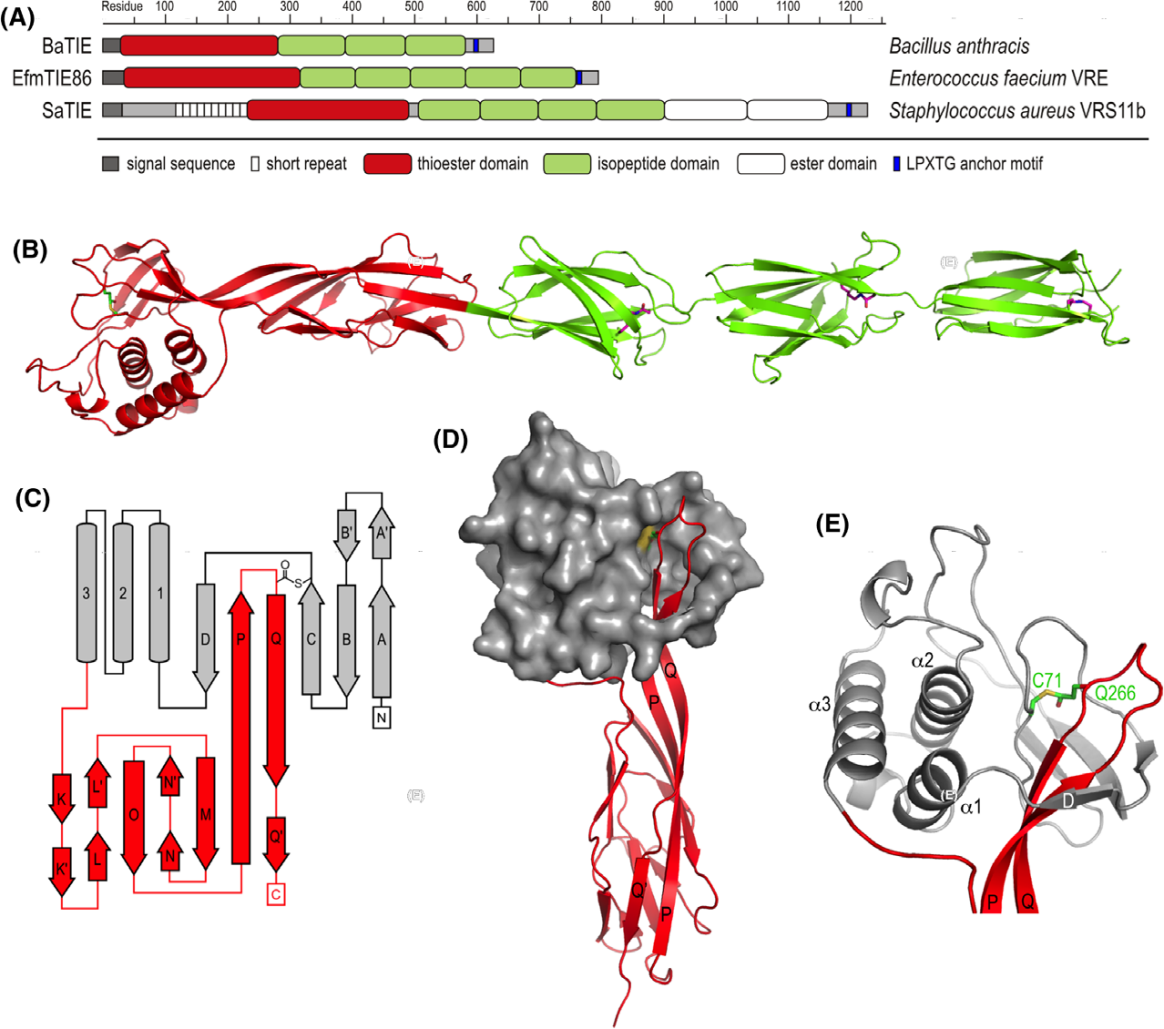
Three TIE proteins containing Class II TEDs. (A) TIE protein domain organization with confirmed and predicted boundaries of TEDs and other modules. (B) Crystal structure of BaTIE. Domains are colored as in (A); intramolecular thioester and isopeptide bonds are shown as sticks. (C) Topology diagram of BaTIE‐TED, highlighting the slipknot structure formed between the N‐terminal lobe (grey) and the characteristic Class II β‐sandwich insert (red). (D) Surface and cartoon representation of BaTIE‐TED slipknot, colors as in (C); selected secondary structure elements are labeled. (E) Detailed view of the thioester bond‐forming residues in BaTIE‐TED.

We selected three TIE proteins, previously predicted to contain Class II TEDs,^5^ for structural studies. The *B. anthracis* BaTIE and the 86 kDa *E. faecium* EfmTIE86 proteins are predicted to comprise a Class II TED as well as three and five CnaB‐type isopeptide domains (IPDs), respectively.^5^ In addition to a TED and four IPDs, the *S. aureus* SaTIE protein is predicted to also contain two ester domains [Fig. 1(A)]. SaTIE and BaTIE have been experimentally confirmed to contain thioester bonds.^5^ BaTIE was among the first surface proteins from *B. anthracis* described, and has previously been studied as BasC and BA5258.^9^, ^10^, ^11^, ^12^ It has been suggested to function as a collagen‐binding protein.^9^ SaTIE is the only TIE protein so far identified in *S. aureus*. EfmTIE86 was identified in genomes of vancomycin‐resistant *Enterococcus faecium* clinical isolates. The latter two proteins remain uncharacterized.

### *BaTIE: the first full length sortase‐anchored surface protein structure*

A BaTIE construct (Glu35‐Lys557 of the full, translated open reading frame) was designed to exclude both the N‐terminal Sec secretion signal peptide (Met1‐Ala34), and a 38‐residue C‐terminal region upstream of the LPATG putative sorting motif predicted to be natively unfolded. Its experimental molecular mass (58,441 Da) was determined by ESI‐MS as 70 Da lighter than predicted, consistent with the formation of a single thioester bond (evolving one molecule of ammonia) and three isopeptide bonds (evolving two molecules of ammonia and one water molecule).

The crystal structure of BaTIE was determined to 2.58 Å resolution using Se‐SAD phasing and standard refinement approaches (Table 1). Continuous electron density between the Cys71 and Gln266 side‐chains of BaTIE confirms the presence of a thioester bond in this position (Fig. 1), consistent with previous data.^5^ Further, continuous electron density between Lys297/Asn373, Lys384/Asp464, and Lys475/Asn555 side‐chains reflects the presence of isopeptide bonds, positioned adjacent to putative catalytic residues Glu343, Glu443, and Glu524, respectively.

**Table 1:** X‐ray Data Collection and Refinement Statistics

	BaTIE	SaTIE‐TED	EfmTIE86‐TED	EfmTIE86‐TED
	SeMet	SeMet	Native	SeMet
*Data collection*				
Wavelength (Å)	0.9282	0.920	0.97625	0.9795
Space group	*C*121	*P*2_1_2_1_2_1_	*C*121	*P*2_1_2_1_2
Molecules in ASU	2	1	4	1
Cell dimensions				
*a*, *b*, *c* (Å)	208.82, 66.5, 106.58	44.03, 73.61, 74.65	197.70, 33.73, 206.05	101.95, 219.69, 33.72
*α, β, γ* (°)	90, 116.81, 90	90, 90, 90	90, 105.878, 90	90, 90, 90
Resolution (Å)^a^	93.19–2.58 (2.65–2.58)	74.99–2.25 (2.32–2.25)	198.19–2.14 (2.18–2.14)	50.99–2.53 (2.57–2.53)
*R*_merge_	6.40 (77.8)	13.3 (106.7)	7.4 (47.4)	26.3 (293.5)
Mean *I*/σ(*I)*	13.1 (1.2)	20.5 (3.3)	9.9 (2.2)	13.2 (1.1)
Completeness (%)				
Overall	99.0 (98.4)	99.8 (97.8)	93.7 (98.0)	89.3 (47.6)
Anomalous	94.0 (90.1)	99.7 (96.8)		89.5 (48.8)
Redundancy				
Overall	3.7 (3.3)	26.8 (21.1)	3.2 (3.1)	24.5 (21.3)
Anomalous	1.9 (1.8)	14.2 (11.0)		13.0 (10.7)
CC(1/2) (%)	100 (60.0)	99.9 (87.3)	99.5 (81.8)	99.7 (61.3)
*Phasing*				
Number of Se sites	5	4		3
FOM pre‐/post‐density modification	0.25/0.49	0.35/0.53		
FOM acentric/centric				0.29/0.18
Phasing power				0.751
autoSHARP final score				2.49
*Refinement*				
Resolution (Å)	93.18–2.58 (2.65–2.58)	74.65–2.25 (2.32–2.25)	198.2–2.14 (2.18–2.14)	
No. reflections	38916	11395	65791	
*R*_work/_ *R*_free_	22.3/25.6 (35.2/37.2)	20.4/24.6 (31.3/28.6)	23.5/25.9 (32.3/31.3)	
No. of non‐hydrogen atoms				
Protein	8164	1882	8594	
Ligand/ion/water	0/7^b^/55	4^c^/2^b^/61	19^d^/0/334	
Wilson B‐factors (Å^2^)	58.2	34.0	34.8	
Average B‐factors (Å^2^)	78.0	45.0	42.0	
RMSD bond lengths (Å)	0.01	0.01	0.01	
RMSD bond angles (°)	1.14	1.15	1.34	
MolProbity Score	0.70 (100th percentile)	0.91 (100th percentile)	0.61 (100th percentile)	

a The highest resolution shell is shown in parenthesis.

b Most probably zinc ions.

c Acetate.

d Glycerol and tetraethylene glycol.

The structure reveals BaTIE is a linear array of a TED and three IPDs (IPD1–IPD3), measuring approximately 20 nm from end‐to‐end [Fig. 1(B)].

**Figure 2:**
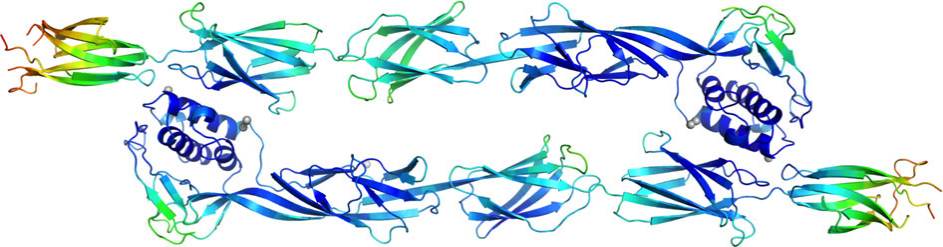
Asymmetric unit of BaTIE crystals comprising two monomers. The rainbow color gradient represents atomic B factors, increasing from blue to red. Zinc ions are shown as grey spheres.

In both chains of BaTIE in the asymmetric unit (ASU), the electron density for IPD3 gets progressively weaker the farther away from IPD2 the residues are positioned, suggesting flexibility of IPD3 in the crystal. For the residues furthest away from IPD2, there is significant ambiguity of side‐chain positions, and poor confidence of backbone tracing (residue 529 of the A chain and residues 528–529 and 557 of the B chain were not modeled). The increase in flexibility of IPD3 coincides with increased temperature factors relative to the rest of BaTIE (Fig. 2).

The BaTIE ASU contains seven bound metal ions in three unique sites. Two of these are formed by side‐chains belonging to the two molecules in the asymmetric unit and water molecules (Site 1: chain A, Glu112, His115, chain B Glu513, one water; Site 2: two ions, chain A Asn69, chain B Glu377, three waters). The third site is coordinated by Asp142 and Gln144 side‐chains belonging to two neighboring ASUs, and two water molecules. It is likely these are zinc ions derived from the crystallization condition and are unlikely to be biologically significant, given that crystal packing is a prerequisite for co‐ordination.

### *Three crystal structures representing the class II TED fold*

The structure of BaTIE enabled the informed design of expression constructs for two further Class II TEDs. SaTIE‐TED (Gln254‐Gly502) and EfmTIE86‐TED (Asp39‐Ala314) were determined by ESI‐MS to be 17 Da lighter than predicted (suggesting the presence of a thioester bond), and readily yielded high‐quality crystals. SaTIE‐TED and EfmTIE86‐TED crystal structures were determined using Se‐SAD phasing and standard refinement procedures (Table 1). Continuous electron density between Cys and Gln side‐chains either previously shown or predicted to form thioesters (SaTIE‐TED: Cys296 + Gln467,^5^ EfmTIE86‐TED: Cys88 + Gln292) confirmed the presence of these bonds.

**Figure 3:**
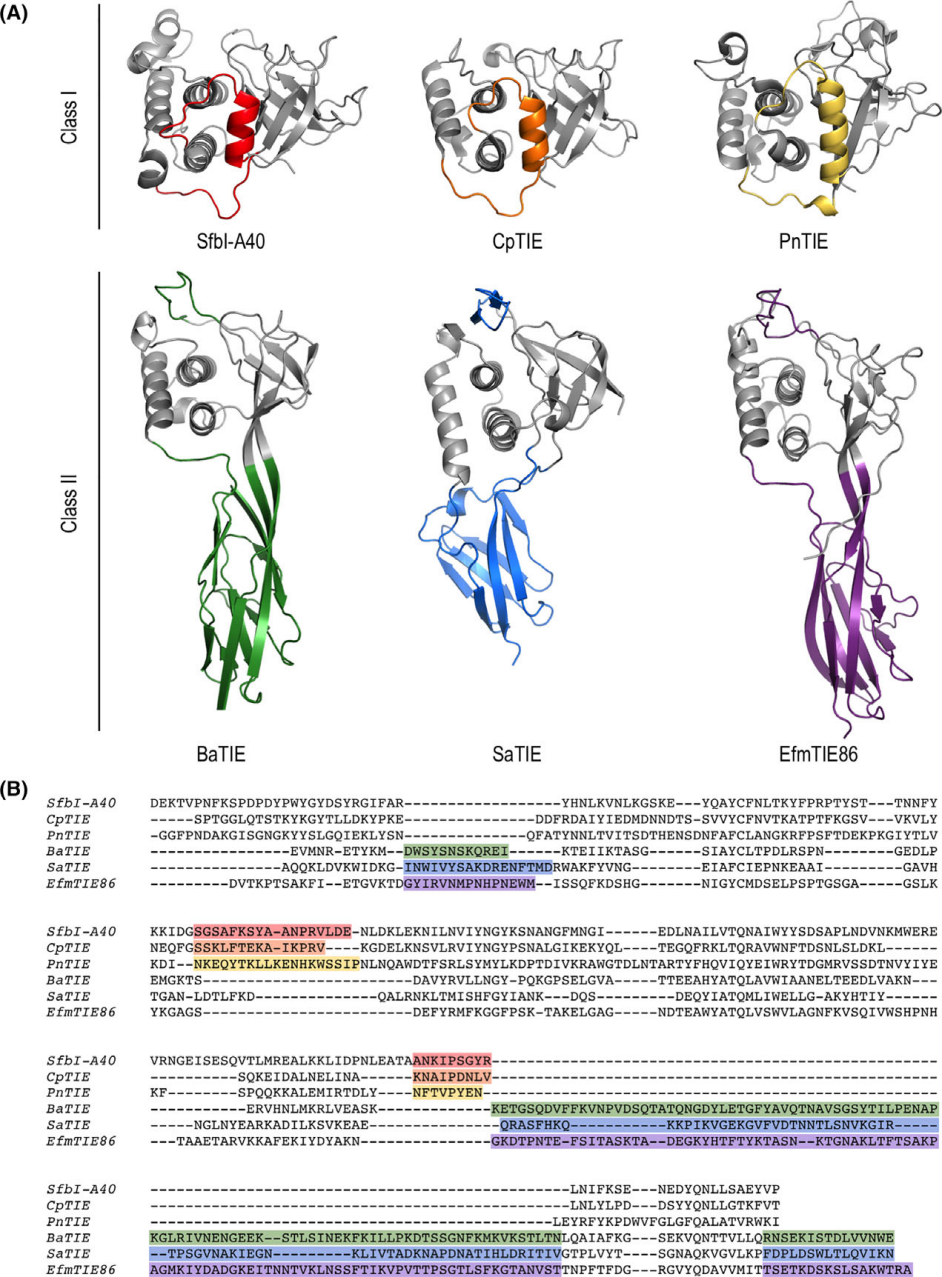
Structural basis of TED classification. (A) Three Class I TEDs (PDB accession codes 5A0L (SfbI‐A40), 5A0G (CpTIE), 5A0N (PnTIE)^5^) and three Class II TEDs are shown in cartoon representation. Structural elements that define the two classes are indicated by colors. (B) Class‐defining indels are highlighted in a structure‐based sequence alignment, colored as in (A).

Despite pairwise identities of 18–23%, all three Class II TEDs in this study display very similar tertiary structures (Fig. 3). Searches with the DALI^13^ protein comparison server highlight the closest homolog as the Class I TED of the *Clostridium perfringens* CpTIE protein (PDB entry 5A0G), which is 21%, 12%, and 15% identical to BaTIE‐TED, SaTIE‐TED, and EfmTIE‐86‐TED, respectively. The RMSD and DALI Z scores for aligning CpTIE‐TED to the three Class II TEDs are 2.6 Å, 13.0; 3 Å, 13.4; and 2.8 Å, 13.2. The structural similarity is limited to the upper lobe of Class II TEDs. All other homologs identified by DALI were proteins containing immunoglobulin‐like folds that match the TED β‐sandwich domain, but not the upper lobe. While the subdomains of Class II TEDs resemble known protein folds, their combination in one‐fold is novel.

**Figure 4:**
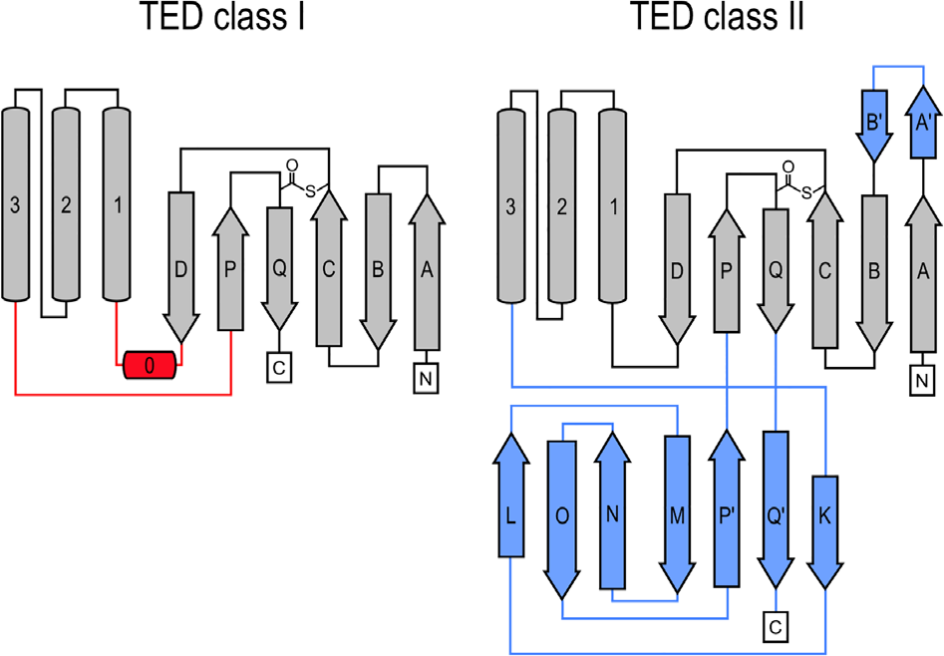
Topology diagrams for SfbI‐TED and SaTIE‐TED, representative of TED Classes I and II, respectively. Secondary structure elements are labeled with equivalent numbers (α‐helices) and letters (β‐strands) to reflect features conserved in both classes. Unique features in either class are highlighted in red and blue. Thioester bonds are also indicated.

The upper lobes of the Class II TEDs correspond to canonical Class I TED folds, comprising a six‐stranded antiparallel β‐barrel and a three‐helix bundle. However, the Class II TEDs lack an α‐helix (α0) that, in Class I TEDs, connects β‐strand D and α1. Interestingly, α0 coincides with the first indel identified in the TED alignment,^5^ (Figs. 3 and 4). A more significant and defining difference between Class I and II TED folds is the replacement of an approximately 10‐residue linker between α3 and β‐strand P in Class I TEDs with a seven‐stranded β‐sandwich. This domain is formed almost entirely of an approximately 75‐residue insertion, coinciding with the second indel previously identified (Figs. 3 and 4). In both TED classes, the thioester bond‐forming Cys is contributed by β‐strand C, and the bond‐forming Gln is provided by β‐strand Q. In Class II TEDs, β‐strands Q and P form an extended, highly twisted β‐hairpin that loops back through the N‐terminal lobe to complement the β‐barrel subdomain, forming a slipknot‐like structure [Figs. 1(C–E) and 4]. A domain insertion topology has also been reported for the thioester protein Cpa in which a Class I TED fold is inserted into an isopeptide domain.^3^ However, in contrast to Cpa, in Class II TEDs the thioester fold is interrupted by an immunoglobulin domain. In the context of the mature TIE protein, insertion of the immunoglobulin fold projects the TED by an additional 50 Å away from the bacterial surface, and provides a link to the remaining stalk. It is interesting to speculate how the slipknot structure may change upon covalent receptor recognition, and whether this has further functional role, for instance, in mechanical stability of a covalent TED complex.

**Figure 5:**
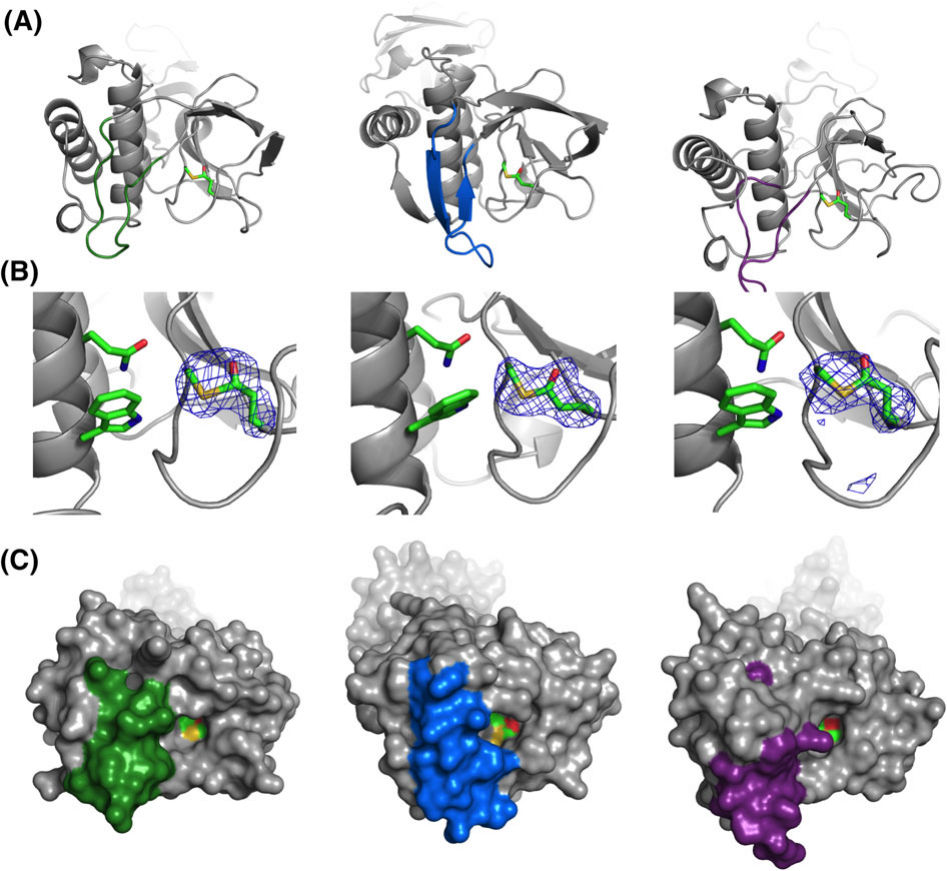
Class II TED structural details. (A) Cartoon representations of Class II TEDs highlighting the location of the specificity loops (colored green, blue and purple) in relation to the thioester bonds (represented as sticks colored green, red, yellow). (B) Zoom‐in of the thioester bonds in Class II TEDs, including the position of the Gln and Trp residues of the TQXXΦW motif, all shown as sticks. Thioester bonds are shown as sticks with *F*_obs_–*F*_calc_ omit density superimposed, contoured at 2.5σ (EfmTIE86‐TED) and 3σ (BaTIE‐TED, SaTIE‐TED). (C) Surface representations of the three Class II TEDs showing thioester access pores, partially defined by specificity loops (green, blue, and purple). Atoms from the thioester bond forming residues are mapped to the surface (colored as in (A)).

As observed for Class I, Class II TEDs have conserved Gln and Trp residues positioned on α2 directly adjacent to the thioester bonds [Fig. 5(B)]. With exception of EfmTIE86‐TED Trp146, side‐chains of these residues hydrogen‐bond with the thioester‐forming Cys backbone. The Gln/Trp motif in Class II TEDs diverges slightly from the TQXXΦWXΦXζ motif defined for Class I, which is redefined here as TQXXΦW, accounting for a lack of conservation after the Trp across both classes.

Receptor access to the thioester bond is restricted by two structural features (Fig. 5); the first is a loop between β‐strands A and B, positioned adjacent to the cleft between the β‐barrel and helical subdomains. Although supporting experimental evidence is currently lacking, this region, termed the specificity loop, is hypothesized to contribute to substrate specificity.^5^ In Class II TEDs, this loop is extended by approximately 15 residues, and covers the cleft between the β‐barrel and α‐helical subdomains (Figs. 3 and 5). The specificity loop also contributes to forming a pore over 8 Å deep, the second restriction on access to the thioester bond [Fig. 5(C)]. The depth of this pore is longer than, for example, a Lys side‐chain (7 Å), strongly suggesting that in order for the thioester to interact with its cognate receptor, the surrounding tertiary structure must undergo a conformational change. Such a change could be initiated by recognition events around the specificity loop, potentially resulting in the displacement of β‐strands P and Q from the N‐terminal lobe. This may facilitate intermolecular isopeptide bond formation between the thioester carbonyl group and a receptor nucleophile, such as a Lys side‐chain. The hypothesis that such a structural rearrangement may be essential for lasting bond formation between a TED and its cognate receptor has recently been supported by force microscopy of the C‐terminal TED from Cpa.^14^

### *BaTIE does not bind to collagen*

BaTIE, or BA5258, was previously reported to bind to bovine collagen Type I.^9^ In this previous study, rBA5258 (Asn38‐Leu371) comprised the entire TED plus most of the first IPD, but terminated two residues before isopeptide bond‐forming Asn373. Its reported experimental mass agreed with its theoretical mass,^9^ suggesting an intramolecular thioester bond was not formed, possibly due to aberrant protein folding induced by long‐range effects of the incomplete IPD.

**Figure 6:**
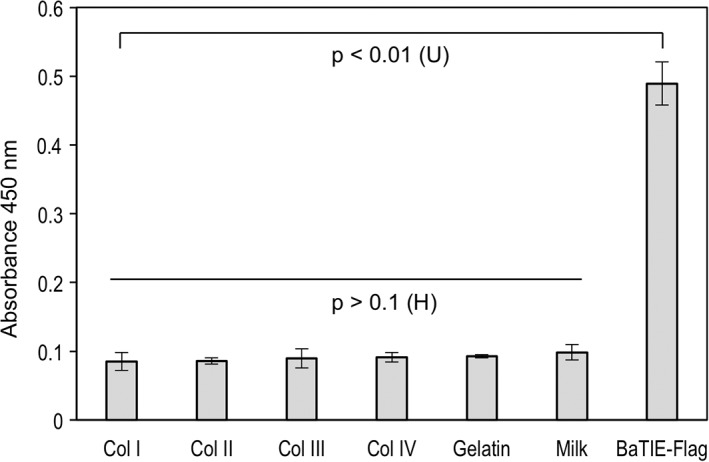
BaTIE does not bind to collagens. BaTIE binding to Collagens I–IV (Col I–IV) and gelatin are shown. For Col IV, gelatin, and BaTIE‐Flag *n* = 5, for all others *n* = 6. *P* values were derived from Kruskal–Wallis *H* test, or Mann–Whitney *U* test, indicated by H and U, respectively.

To test collagen binding of BaTIE in its full‐length, enzyme‐linked immunosorbence assays were performed using a BaTIE‐Flag tag fusion. These static adhesion assays strongly suggest that BaTIE does not interact with Collagens I–IV or gelatin (Fig. 6). Our experiments suggest that the previously reported collagen‐binding activity for BaTIE was not thioester‐mediated and may have been an artifact of rBA5258 design, giving rise to non‐specific binding through exposed hydrophobic core residues. Therefore, the biological target of BaTIE remains unknown.

## Conclusions

Sequence similarity searches suggest that TEDs and TIE proteins are extremely abundant, and very diverse with respect to their domain composition and sequences. Multiple sequence alignment suggests that there are only two classes of TEDs. However, given the rapid increase in available genetic information, other structurally distinct TED classes might emerge. The three structures presented here provide a structural basis for the differentiation of two TED classes, and will aid identification of new TEDs. It is currently unclear if the structural features that distinguish TED classes have functional significance, that is, if they differ systematically in terms of reactivity, receptors, or biological role. In this regard, it is interesting to note that some genera of Gram‐positive bacteria encode exclusively Class I or Class II TEDs. For instance, streptococcal thioester proteins only contain Class I TEDs (e.g., *S. pyogenes* SfbI, FbaB, Cpa), whereas *Enterococcus* and *Bacillus* encode only Class II.

## *Materials and Methods*

### *Cloning and expression*

BaTIE, SaTIE‐TED, and EfmTIE86‐TED constructs were produced through traditional cloning techniques, with NcoI and BamHI restriction sites incorporated at insert 5′ and 3′ ends, respectively. DNA encoding SaTIE‐TED (Gln254‐Gly502) was amplified from genomic DNA as described previously.^5^ EfmTIE86‐TED (Asp39–Ala314) was amplified from genomic DNA and corresponds to UniProt entry A0A1A7T0E1. BaTIE (Glu35–Lys557) was amplified from a synthetic gene created by Biomatik (Canada) based on UniProt entry A0A0F7RA58. Amplified DNA was inserted into pEHisTEV.^15^

BaTIE‐Flag was produced through two rounds of PCR using the BaTIE forward oligonucleotide with reverse oligonucleotides “FlagA” and “FlagB,” and consists of the BaTIE sequence followed directly by the primary sequence “DYKDHDGDYKDHDIDYKDDDDK.” The presence of the FLAG‐tag was confirmed by MALDI‐TOF with MS/MS. These primers were used, with restriction sites highlighted in bold:

**Table:** 

BaTIE forward	CGCCGC**CCATGG**AAGTAATGAACAGGG
BaTIE reverse	CCGGG**GGATCC**CTATTTCTTATTTTTCAC
FlagA reverse	CACTC**GGATCC**TTTATAATCACCGTCAT
	GGTCTTTGTAGTCTTTCTTGTTTTTCAC
FlagB reverse	GTT**GGATCC**TTACTTGTCATCGTCATCCTTGT
	AATCGATGTCATGATCTTTATAATCACCGTC
SaTIE‐TED forward	CACAG**CCATGG**CGCAACAAAAATTAGAT
SaTIE‐TED reverse	CT**GGATCC**CTACCCTGTTTCAGTATCTTC
EfmTIE86‐TED forward	CCCGGG**CCATGG**GGGATGTCACAAAACCG
EfmTIE86‐TED reverse	CCCC**GGATCC**CTAAGCTCTTGTCCATTTA

### *Protein expression and purification*

Proteins were produced in *E. coli* BL21 (DE3) grown in Luria Broth at 37°C until A_600_ 0.6–0.8. Expression was induced with 1 mM isopropyl 1‐thio‐β‐d‐galactopyranoside and cultures incubated at 16–25°C for 16–20 h. Selenomethionine (SeMet) incorporation was achieved using SeMet minimal media (18.7 mM ammonium chloride, 14.7 mM monopotassium phosphate, 22.4 mM disodium phosphate, 5%(w/v) glycerol, 1.1 g/L glucose‐free nutrient mix (Molecular Dimensions; MD12–502‐GF), containing vitamins and amino acids excepting l‐methionine, and 50 μg/mL kanamycin, pH 7.4). Bacterial cultures were incubated at 37°C for 15 min, then supplemented with 60 mg/L SeMet (Acros Organics). Incubation thereafter continued until OD_600_ 0.6–0.8, after which 100 mg/L of each Lys, Phe, Thr, and 50 mg/L of each Ile and Val were added in order down‐regulate *de novo* synthesis of Met and drive incorporation of SeMet. After an additional 20 min incubation at 37°C, expression was induced with 1 mM IPTG and the temperature lowered to 16°C; cells were incubated for 18–24 h before harvesting. All proteins were expressed with a cleavable N‐terminal His_6_‐tag. Cell pellets were re‐suspended in phosphate‐buffered saline (PBS) (pH 6.0 or 7.2) supplemented with one EDTA‐free protease inhibitor cocktail tablet (Roche, Welwyn Garden City, UK) and 1 mg DNase I per 50 mL of buffer. Cells were lysed with a cell disruptor (Constant Systems, Daventry, UK). Clarified lysate was applied to a Ni^2+^‐IMAC column (GE Healthcare, Deutsch, UK), columns washed with 10 column volumes of buffer (as above) supplemented with 30 mM imidazole, and bound proteins step eluted with buffer supplemented with 300 mM imidazole. Fractions containing TIE proteins were desalted by dialysis and incubated with tobacco etch virus protease (1:20, w/w) at 4°C for 16–20 h. Cleaved proteins possess the non‐native sequence “GAMA” (SaTIE‐TED), “GAMG” (EfmTIE86‐TED), or “GAM” (BaTIE, BaTIE‐Flag) at their N‐termini remaining from the protease and endonuclease recognition sites. Samples were thereafter reapplied to a Ni^2+^‐IMAC column. Cleaved proteins collected in the flow through, which was concentrated and injected onto a HiLoad 16/60 Superdex 75 gel filtration column (GE Healthcare, Daventry) pre‐equilibrated in either 50 mM HEPES (pH 7.0), 150 mM NaCl, 0.02%(w/v) NaN_3_ or 50 mM MES (pH 6.0), 150 mM NaCl, 0.02%(w/v) NaN_3_. SeMet‐substituted protein buffers were supplemented with 2 mM β‐mercaptoethanol. Fractions containing purified TIE proteins were concentrated to 15–30 mg/ml or 15 mg/ml for enzyme‐linked immunosorbent assays (ELISAs). Protein concentration was determined by A_280_ using UV spectrophotometry, and calculated based on theoretical extinction coefficients (ProtParam tool, ExPASy, RRID:SCR_015894).

### *Mass spectrometry analyses*

For intact mass determination, protein samples (20–30 μl, 20–30 μM) were desalted through an XTerra MS C8 2.1 × 10 mm HPLC column (Waters, Milford, MA) and eluted with an increasing acetonitrile gradient (2% (v/v) to 98% (v/v) acetonitrile in aqueous 1%(v/v) formic acid) and delivered into an electrospray ionization MS (LCT, Micromass, Manchester, UK) previously calibrated with a myoglobin standard. Multiply charged signals and their time of flight were obtained and deconvoluted using MaxEnt1 software.

For protein identification, peptide mass fingerprinting of tryptic in‐gel digests was performed using a 4800 MALDI TOF/TOF analyzer (Applied Biosystems, Foster City, CA). Protein/peptides (0.5 μl) were mixed with 0.5 μl alpha‐cyano‐4‐hydroxycinnamic acid matrix (10 mg/ml in 50:50 acetonitrile:0.1% trifluoroacetic acid) and left to dry on a MALDI plate. MALDI TOF/TOF data were analyzed using GPS Explorer (ABSciex, Warrington, UK) to interface with the Mascot 2.4 search engine (Matrix Science, London, UK).

### *Crystallization, data collection, structure determination, and refinement*

BaTIE SeMet crystals were obtained from protein purified in HEPES buffer, pH 7.0, and concentrated to 20 mg/mL. Crystals were grown through sitting drop vapor diffusion at 20°C by combining protein and precipitant (0.1 M sodium cacodylate pH 6.5, 70 mM zinc acetate, 10–12%(w/v) polyethylene glycol (PEG) 6000, 2%(v/v) methanol) in a 2:1 ratio. X‐ray datasets were collected from crystals cryoprotected by brief incubation in reservoir supplemented with 30%(v/v) glycerol using a micro‐focus beamline.

SaTIE‐TED SeMet and native crystals were obtained from protein purified in MES buffer, pH 6.0, and concentrated to 30 mg/mL. Crystals were grown through hanging drop vapor diffusion at 20°C by combining protein and precipitant (25%(w/v) PEG 2000 monomethyl ether (MME), 0.1 M Tris pH 7.5, 0.1 M zinc acetate) in a 1:1 ratio, and were cryoprotected with mother liquor supplemented with 20%(w/v) PEG 2000 MME.

EfmTIE86‐TED SeMet and native crystals were obtained from protein purified in HEPES buffer, pH 7.0, and concentrated to 15 mg/mL. Crystals were grown through hanging drop vapor diffusion at 20°C by combining protein and precipitant (0.1 M sodium citrate pH 5.0, 50 mM (NH_4_)_2_SO_4_, 24.62% (w/v) PEG 4000) in a 1:1 ratio, and were cryoprotected with mother liquor supplemented with 20% (v/v) glycerol.

Diffraction data were collected at the Diamond Light Source (UK) on beamlines i03 (EfmTIE86‐TED), i04–1 (BaTIE, SaTIE‐TED). Data were processed using Xia2 (RRID:SCR_015746)^16^ except for EfmTIE86‐TED native data, processed with autoPROC (RRID: SCR_015748).^17^ All structures were solved by Se‐SAD phasing. For BaTIE and SaTIE‐TED, data were input into the Crank2 pipeline in the CCP4 suite, which also built the initial model.^18^, ^19^ For EfmTIE86‐TED, data were input into autoSHARP^20^ by the i03 data processing pipeline, which outputs a poly‐Ala model. This model was input into Buccaneer (RRID:SCR_014221),^21^ and the resulting model was used as a search model in Phaser (RRID:SCR_014219) to apply phases to the native dataset.^22^

Final models were produced through iterative rounds of refinement using REFMAC5 (RRID:SCR_014225)^23^ and manual rebuilding with Coot (RRID:SCR_014222).^24^ Non‐crystallographic symmetry restraints were used during BaTIE and EfmTIE86‐TED model refinement. Translation‐Liberation‐Screw (TLS) restraints were applied in each case. For BaTIE, each TED and IPD was defined as a TLS group. For SaTIE‐TED, the upper Class I subdomain and the lower β‐barrel were treated as separate TLS groups; and for EfmTIE86‐TED, the upper lobe subdomain, β‐strand K, and β‐strands L–Q were treated as separate TLS groups. Structure validation was performed using MolProbity (RRID:SCR_014226)^25^ and Coot. Data collection, phasing, and refinement statistics are shown in Table 1.

### *Collagen binding assays (ELISA)*

Immuno 96‐well plates (Thermo Fisher Scientific, London, UK) were coated with rat collagen I (Sigma, Gillingham, UK), bovine collagen II (MDB Biosciences, Oakdale, MN), human collagen III (Sigma), human collagen IV (Sigma), and porcine gelatin (VWR, UK) at 10 μg/mL, and BaTIE‐Flag at 5 μg/mL, in 0.01 M acetic acid for 2 h at room temperature. Wells were washed three times with 1% (w/v) non‐fat milk dissolved in phosphate‐buffered saline (PBS) containing 0.1%(v/v) Tween‐20 between each incubation step. All incubations were performed for 1 h at room temperature. Wells were blocked with 5% (w/v) non‐fat milk dissolved in PBS containing 0.1% (v/v) Tween‐20, and BaTIE‐Flag applied to wells at 5 μg/mL dissolved in adhesion buffer. Mouse anti‐DDDDK tag antibodies (Abcam, Bristol, UK) were added at a dilution of 1:20,000 in adhesion buffer prior to addition of the TMB substrate system (Thermo Fisher Scientific). Chromogenic output was detected at 450 nm.

In each group, data points greater than the first quartile plus 1.5 times the interquartile range (IQR) or less than the third quartile minus 1.5 times IQR were excluded as outliers. In each Collagen IV, gelatin, and BaTIE‐Flag datasets, one outlier was identified and excluded; therefore, sample sizes of these groups are *n* = 5. For all others, all data points were included (*n* = 6). A Kruskal–Wallis *H* test shows there is a statistically significant difference in A_450_ among all ligands tested, *χ* ^2^ 7 = 18.4, *P* < 0.01 with mean rank scores of 92 for Collagen I, 92.5 for Collagen II, 115.5 for Collagen III, 114.5 for Collagen IV, 134 for gelatin, 181 for milk, and 215 for BaTIE‐Flag. However, after exclusion of the BaTIE‐Flag binding data, this same test indicates that there is no significant difference among the remaining datasets (*χ* ^2^ 6 = 10.6, *P* > 0.1). A Mann–Whitney *U* test indicates A_450_ is greater for BaTIE‐Flag than for Collagen I (U 9 = 0, *P* < 0.01), and by extension all other ligands tested.

## *Accession Codes*

Protein structures, and the data used to derive these, have been deposited at the PDBe (RRID:SCR_004312) with accession numbers 6FWV (BaTIE), 6FX6 (SaTIE‐TED), and 6FWY (EfmTIE86‐TED).
